# Leading healthcare improvement: an implementation strategy

**DOI:** 10.1186/s12913-026-15046-4

**Published:** 2026-06-30

**Authors:** Kelley Arredondo, Anthony Ecker, Lindsay Vaclavik, Jacquelene Shahin, Ashley Thompson, Molly Horstman, David Ramsey, Kyler M. Godwin

**Affiliations:** 1 Houston VA HSR&D Center for Innovations in Quality, Effectiveness, and Safety, Michael E. DeBakey VA Medical Center, MEDVAMC 156, 2002 Holcombe Blvd., Houston, TX USA; 2 Department of Medicine, Section of Health Services Research, Baylor College of Medicine, Houston, TX USA; 3 VHA Office of Rural Health’s Veterans Resource Center in White River Junction, White River Junction, VT USA; 4 VA South Central Mental Illness Research, Education, Clinical Center, a Virtual Center, Houston, TX USA; 5 Menninger Department of Psychiatry and Behavioral Sciences, Baylor College of Medicine, Houston, TX USA

**Keywords:** Quality improvement, Leadership training, Change management, Interprofessional teams, Implementation strategy

## Abstract

**Introduction:**

Healthcare professionals often implement quality improvement (QI) initiatives but lack training to implement and sustain QI initiatives. Leading Healthcare Improvement (LHI) is a course that combines principles from change management, team science, and leadership effectiveness into an implementation strategy to enable clinicians to lead sustainable change.

**Methods:**

LHI is a 6-session, 4-month interactive training. Learners work on an active QI project throughout the course. A Wilcoxon signed-rank test was conducted to evaluate pre- and post-course changes in learners’ self-reported confidence across five QI competencies.

**Results:**

Veterans Healthcare Administration clinicians and fellows completed the LHI pre- (*n* = 111) and post-course (*n* = 74) evaluations with 70 responding to both evaluations. The 70 learners self-reported significantly increased confidence after the training in each of the QI competencies: communicating urgency (W = 56, *p* < 0.001), developing and leading interprofessional teams (W = 48, *p* < 0.001), creating and communicating project vision (W = 54, *p* < 0.001), developing strategies to remove barriers (W = 60, *p* < 0.001), and communicating project impacts to leadership (W = 62, *p* < 0.001).

**Discussion:**

LHI is an implementation strategy curriculum intended to support healthcare professionals in leading system-level changes. Its emphasis on key partner influence, interprofessional teamwork, and real-time project application may help address gaps in existing training and has the potential to strengthen capacity for sustained healthcare improvement.

**Supplementary Information:**

The online version contains supplementary material available at https://doi.org/10.1186/s12913-026-15046-4.

## Introduction

Healthcare professionals who are trained in leadership principles [1, 2] and adept with implementation strategies [3] are better equipped to translate clinical best practices within their local healthcare context, effectively manage interpersonal relationships, and cultivate partnerships needed to spread and sustain healthcare improvement throughout a healthcare system. However, healthcare professionals are rarely exposed to structured training in these areas during their formal clinical education, despite increasing recognition of their importance.

Traditional change leadership theories, such as Kotter’s model for leading change [4], focus on how formal leaders (i.e., those at the top of an organization) can influence those they manage within a hierarchy (i.e., those at the bottom of an organization) towards desired organizational goals [5]. Many healthcare institutions are largely organized in hierarchies, often with overlap between subsets of the system (e.g., a clinic within a department) [6]. This conventional, top-down approach can suppress individual and team creativity and adaptability within organizations, impeding creative QI solutions and implementation.

Research demonstrates that effective teams and teamwork are positively related to quality of care delivery [7, 8]. Yet, in real-world healthcare settings, numerous competing demands, such as various process or QI interventions simultaneously vying for resources (e.g., personnel, funding, and time), create implementation challenges. Therefore, clinicians must not only navigate complex systems but should also understand individual and social relationships, norms of behavior, and organizational goals to generate urgency for their intervention and garner buy-in from others [9].

The sustainability and diffusion of best clinical practices require theory-based interpersonal leadership skills for effectively managing teams, understanding and motivating people who resist change, and carefully applying the best implementation strategies to the appropriate contexts. New training strategies are needed that translate these theory-based critical skills into one, integrated set of competencies for the practical implementation of healthcare improvement by frontline clinicians. It is imperative to translate QI research into actionable healthcare delivery through implementation frameworks [10]. Although implementation science provides frameworks for translating evidence into practice, many approaches emphasize a singular strategy that may be unrealistic in complex care environments. In contrast, successful interventions in healthcare are often multi-faceted and flexibly apply theories, strategies, and tools to address local needs [11, 12]. While training opportunities exist for implementation science, leadership development, change management and QI, these are often offered as singular approaches that do not address the overlapping and synergistic nature of these fields.

To address this training gap, we developed the Leading Healthcare Improvement (LHI) course that teaches clinicians the LHI implementation strategy. Rather than functioning solely as a traditional leadership development or QI curriculum, LHI integrates core principles from leadership development, change management, QI, and team science into a structured, experiential learning implementation strategy. The course is intentionally designed to support the application of implementation and improvement principles to real-world clinical projects, thereby bridging the gap between didactic training and practice-based improvement efforts. In this way, LHI serves as a hybrid implementation strategy that combines educational and implementation focused components to enhance clinicians’ ability to initiate, lead, and sustain healthcare improvement efforts. A unique contribution of LHI is the emphasis on interpersonal skills with a dual top-down and bottom-up approach to empower both formal and informal leaders to drive sustainable change throughout the institution [13]. Furthermore, LHI requires that learners change their behavior and their traditional way of thinking to successfully implement their change interventions.

This article describes the LHI implementation strategy curriculum as a conceptual and applied framework for clinician-led improvement projects. We provide an overview of theoretical foundations, practical components, implementation context, and share lessons learned. Ultimately, LHI aims to help learners implement change through understanding human behavior and motivation.

## Methods

Baylor College of Medicine IRB deemed the need for informed consent unnecessary according to national regulations (45 CFR 46.104).

### Leading healthcare improvement structure

LHI consists of a series of six, 90-minute, interactive sessions over 4 months that teach implementation science components in a practical and accessible way to a non-research audience. Each session focuses on elements needed to successfully lead healthcare improvement including (1) Generate Urgency, (2) Build Teams, (3) Create Vision and Communicate, (4) Solve Problems and Celebrate Small Wins, and (5) Build Momentum and Embed Change. The sixth session consists of learners’ project presentations, where they present their project progress across the 5 QI elements covered throughout the course (see Table 1 for further information about each session’s learning objectives and tools). Each session is led by a faculty member who has expertise in health professions education as well as experience with implementation science and QI. Faculty also facilitate small group breakouts during each session where learners apply the session materials to their own projects. Learners are expected to participate in large and small group discussions, including turning on their webcams, when possible, and either unmuting themselves to speak or typing into the chat box. These expectations are communicated on the recruitment flyer, a welcome email, as well as throughout the course.

**Table 1:** Leading healthcare improvement course content

Session	Learning Objectives	Tool(s)/Pre-work
Generate Urgency	1. Describe why it is important to create a sense of urgency around a problem 2. Identify key partners for a change project 3. Frame the problem in a manner that gets others to see the problem as their own 4. Apply a threats vs. opportunities matrix 5. Practice developing and communicating a pitch for change	1. Key Partner Analysis Matrix 2. Storytelling Tool 3. LHI Project Template (ongoing use)
Build Teams	1. Explain what effective teamwork entails 2. Identify who should be included in your interprofessional team 3. Understand the importance of roles & responsibilities for teams	1. Team Charter
Create Vision & Communicate	1. Practice a clear, effective vision statement for your QI projects 2. Articulate different modalities for effective communication to key partners 3. Analyze a breakdown in communication	1. Key Partner Analysis Matrix 2. Vision Statement Template
Solve Problems & Celebrate Small Wins	1. Describe power, influence and authority 2. Explain how influence can be used to address resistance 3. Communicate about a change project with a value proposition 4. Understand why creating short term wins is important 5. Differentiate between individual PDSA wins and project milestones 6. Identify opportunities to celebrate short term wins	1. Value Propositions 2. Celebrate Victories
Build Momentum & Embed Change	1. Describe why and how to build on the change in your project 2. Identify barriers and facilitators for your project using the CFIR implementation framework 3. Understand the important role that culture and context play in organizational change 4. Describe strategies for sustaining organizational change 5. Discuss elements of effective leadership within organizational change	1. Barriers & Facilitators
Learners’ Presentations	1. You will be presenting your projects in your breakout groups 2. The presentation template will be provided 3. You will have 10 min to present and will receive 5 min feedback	1. Presentation Template

LHI is open to individuals within the Veterans Affairs (VA) with an intermediate to advanced knowledge of QI and who have an existing QI or implementation project, or plan to start one during the course. A recruitment flyer was shared with operational partners to distribute to a broad VA audience via email blasts. The flyer contains a link to an intake form where applicants provide information on their background, prior experience in QI and implementation, and information on their project. Responses are reviewed by the team to ensure applicants have both prior knowledge and a current project they are working on before welcoming them to the cohort.

The need for LHI arose from leading a national training program for advanced fellows in QI and implementation (i.e., VA Quality Scholars Advanced Fellowship program) and appreciating that technical skills in healthcare improvement were not enough to equip learners to lead change through a system. Relational and leadership skills must be incorporated, as well. Thus, LHI integrates principles from leadership development, change management, QI, and team science into a cohesive strategy.

LHI was designed to be delivered virtually and was delivered virtually throughout the study period (Fall 2022-Spring 2024). Program structure and delivery format remained consistent between Fall 2022 and Spring 2024 despite the COVID-19 pandemic.

Designed to fill a persistent training gap for clinicians tasked with leading implementation efforts, LHI offers a structured yet adaptable educational experience that supports real-time application and team-based learning. The LHI implementation strategy provides learners with accessible tools and methods to lead change through a system. LHI trains healthcare clinicians, including those with responsibilities for training residents and students, and operational staff to lead healthcare improvement to successfully navigate their organizational context, promote change by influencing key partners and implement effective clinical practices.

The LHI training hub developed several enduring products to support the LHI course, including 9 separate tools. The hub has also developed 8 professionally created flipped classroom videos that are used to provide course content so that the synchronous sessions can primarily focus on one-on-one coaching. This infrastructure is intended to support broader dissemination and scalability, allowing adaptation of LHI content across different institutional and learner contexts, including faculty development, team-based learning initiatives, and operational improvement efforts.

Learners complete self-guided tools on each step of their project prior to each session. These tools are embedded in a structured implementation roadmap that scaffolds learners’ progress and reinforces the course content across sessions. Each session has at least one accompanying tool for the learners to complete and bring to the session. The LHI tools were developed through an iterative process with end-users who had existing QI projects and provided feedback on the clarity and utility of the tools. The LHI strategy targets the individual learner, or implementation practitioner, and individuals on the same team. Because coaching is an integral part of the strategy, learners receive coaching on their projects during each session, and the coaching may be tailored to individuals or teams, depending on the need. This iterative coaching model is designed to promote psychological safety, reinforce practical skill application, and support behavior change.

The course is funded by the VA Quality Enhancement Research Initiative and is one of the initial four funded Implementation Training Hubs. The LHI course has received funding since 2018, signaling the value of LHI as a clinician development initiative with implications for system-wide improvement. Learners consist of clinical and operations leaders as well as research coordinators and fellows. Learners in LHI are required to have some prior experience and/or training in QI and have an active implementation project to work on throughout the course to apply concepts taught in real time. Sessions are delivered via a web-based platform. To facilitate interaction and engagement throughout the web-based sessions, principles of adult learning theory and active-learning strategies [14, 15] are employed during each session.

### Survey

Evaluations were collected across 5 cohorts between Fall 2022 and Spring 2024 using VA’s Research Electronic Data Capture (REDCap) [16] online platform, including a baseline survey and an exit survey focusing on changes in knowledge and self-reported confidence (see Supplemental files 1 and 2). Learners rated their confidence on a 7- point Likert scale (1 = none, 2 = very low, 3 = low, 4 = moderate, 5 = high, 6 = very high, 7 = extremely high) across 5 QI competencies:

1. Communicate the urgency of a QI project to key partners,
2. Develop and lead effective interprofessional teams,
3. Create and communicate project vision,
4. Develop strategies to remove barriers for project implementation and success,
5. Communicate project impacts to leadership.

The baseline and exit surveys also capture the stage of the learners’ projects at the start of the course versus the end, including brainstorming, aim development, building teams, developing measures, pilot testing, implementation/data collection, data analysis, evaluation/Plan Do Study Act, and dissemination. These stages serve as a proxy for project maturation and practical application of course content. Measures were based on learning objectives of the LHI course. They were not excerpted from an existing scale. They were piloted with learners and faculty and revised for clarity based on feedback. Items were analyzed individually and are not part of a broader scale.

We conducted a retrospective pre-post assessment of the impact of LHI training on learners’ self-reported confidence across the 5 QI competencies. We ran Wilcoxon signed-rank tests using Stata [17] to compare pre- and post-training ratings on the 7-point Likert scale given the paired study design and ordinal nature of the Likert-scale data. Pre- and post-course survey responses were matched at the individual level using participant names, which were collected on both surveys for the purpose of longitudinal linkage. After matching was completed, identifiers were removed and analyses were conducted on a de-identified dataset. Learners who took the LHI course and completed both the pre- and post-course surveys were included in the analyses. Learners who only completed the pre-test or the post-test were excluded. Effect sizes (r) were calculated using the formula r = $\:\frac{z}{\surd\:N}$ to estimate the magnitude of change.

## Results

A total of 111 learners completed the pre-evaluation, and 74 learners completed the exit survey, of which 70 completed both evaluations. Only learners who completed both pre- and post-survey evaluations were included in analyses. There were no systematic differences between those that completed just the pre-survey (*n* = 18) versus those that completed both the pre- and post-survey (*n* = 70; see Table 2). The 70 learners were interprofessional and had diverse clinical backgrounds (Table 3). Additionally, they had different roles within the project, with more than half of learners serving as team lead on their projects and about a third serving as a team member (Table 3).

**Table 2:** Comparison of medians and interquartile range of learners who responded only to the pre-survey versus those that responded to both pre- and post-surveys

	Pre-only (*N* = 18)	Paired Responses (*N* = 70)	*p*-value
	Median (IQR)	Median (IQR)	
Communicate the urgency of a QI project to key partners	4 (4–5)	4 (3–5)	0.563
Develop and lead effective interprofessional team	4 (4–5)	4 (4–5)	0.620
Create and communicate project vision	4 (4–5)	4 (4–5)	0.426
Develop strategies to remove barriers for project implementation and success	4 (3.75-5)	4 (3–4)	0.135
Communicate project impacts to leadership	4 (3.75-5)	4 (3–4)	0.252

Note. IQR = interquartile range IQR, 25th–75th percentile

**Table 3:** Learner (*N* = 70) descriptive characteristics

Variable	*n* (%)
Profession, multiple responses allowed (*N* = 70)	
Administrator/Operations	4 (5.7)
Clinical	43 (61.4)
Project/Program Management	2 (2.9)
Research (i.e., Investigator, Analyst)	16 (22.9)
Research Staff (i.e., Research Coordinator, Health Science Specialist)	3 (4.3)
Trainee	1(1.4)
Other	9 (12.9)
Clinical Background among clinical respondents only (*N* = 43)	
Nurse	8(18.6)
Occupational Therapist	1 (2.5)
Pharmacist	3 (7.0)
Physical Therapist	1 (2.5)
Physician	16 (37.2)
Physician Assistant	5 (11.6)
Psychologist	7 (16.3)
Social Worker	1 (2.5)
Speech Language Pathologist	1 (2.5)
Project Role, single response (*N* = 70)	
Team Lead	45 (64.3)
Team Member	24 (34.3)
Key Partner	1 (1.4)

Learners reported high overall appraisal of the program, with 99% (*n* = 69) reporting that the course was good, very good, or excellent. Most learners (97%, *n* = 68) stated they would recommend this course to others. Additionally, learners reported a significant increase in perception of skill in all program competencies, including confidence in ability to communicate the urgency of a project to key partners; create and communicate project vision; develop and lead effective, interprofessional teams; develop strategies to remove barriers for project implementation and success; and communicate project impacts to leadership (see Table 4).

**Table 4:** Pre- post-comparison of learners’ confidence level in quality improvement (QI) competencies

QI Competency	Wilcoxon Statistic	*p* value	Effect Size (*r*)
Communicate urgency of QI project	56	< 0.001	0.79
Develop and lead interprofessional teams	48	< 0.001	0.75
Create and communicate project vision	54	< 0.001	0.81
Develop strategies to remove barriers	60	< 0.001	0.81
Communicate project impacts to leadership	62	< 0.001	0.86

Over the course of the training, learners advanced through the stages of their projects. At the beginning of the LHI sessions, some of the 70 learners had completed brainstorming (69%), aim development (53) and building teams (50%). At the end of the LHI session, most learners had completed brainstorming (82%), aim development (78%), and building teams (64%).

## Discussion

Learners in the LHI course reported increased confidence after the training in each of the course competencies including: communicating urgency, developing and leading interprofessional teams, creating and communicating project vision, developing strategies to remove barriers, and communicating project impacts to leadership. LHI supports healthcare professionals in leading system-level changes. Its emphasis on key partner influence, interprofessional teamwork, and real-time project application may help address gaps in existing training and has the potential to strengthen capacity for sustained healthcare improvement.

A unique contribution of LHI is its emphasis on interpersonal skills with a dual top-down and bottom-up approach to instill meaningful change throughout the institution [13]. The observed gains in confidence related to communication, team leadership, and influencing key partners may reflect LHI’s emphasis on these interpersonal and relational skills. The LHI competencies are central to implementation success, particularly in complex healthcare environments where change depends on engagement across hierarchical levels. The large effect sizes observed suggest that focusing on influence and key partner engagement may be a high-yield component of QI and implementation training. LHI presents learners with strategies to influence key partners and team members to adopt their interventions. This emphasis on influence and interpersonal engagement distinguishes LHI from more technically oriented or compliance-driven QI training approaches and suggests its potential value as both a leadership development tool and an implementation strategy.

A critical component of LHI is that it helps learners understand and develop interprofessional teamwork skills within an interprofessional setting [18]. LHI provides learners with tools to identify appropriate team members and their respective roles and responsibilities relative to the purpose of the QI project. Furthermore, LHI trains learners to understand the value of forming teams with the appropriate mix of members that have the knowledge, skills, abilities, and other factors for successful implementation of QI interventions. Given the complex and collaborative nature of healthcare delivery, these interprofessional competencies are widely recognized as important for project success and for cultivating cultures of collaboration and psychological safety, although these outcomes were not directly assessed in this evaluation.

A large body of work has explored the differences between individuals in their willingness to adopt innovations. The rate of adoption is the relative speed with which innovation is adopted by members of a social system. LHI gives learners the tools and behavioral training to engage with different types of adopters to garner buy-in for their change interventions with the goal of facilitating adoption of their innovations; however, adoption outcomes were not measured in the current evaluation. By operationalizing concepts from the diffusion of innovation theory. LHI supports learners in adapting their messaging and engagement strategies to resonate with both early adopters and more cautious key partners.

While LHI was originally developed within the Department of Veterans Affairs system, its core principles are widely applicable across academic health centers and training institutions. The integration of QI, implementation science, change management, and relational leadership into one cohesive curriculum offers a potentially useful model for advancing faculty development, educational innovation with implications for healthcare system improvement that warrant further study.

The current findings must be considered in light of their limitations. First, this evaluation did not include a comparison group, limiting causal assumptions that LHI was the primary driver of confidence ratings. Second, the outcomes were based on self-report ratings of confidence, rather than observed behavior change, implementation outcomes, or patient- or system-level outcomes. Additionally, while the features of LHI are theoretically aligned with effective implementation practices, the current evaluation assessed participant-reported confidence rather than direct measures of implementation or institutional change. Future work would benefit from comprehensive learning and patient/system impact. Third, high rates of post-survey non-completion were observed, which could influence results. For example, learners with lower confidence may have chosen not to complete the survey. Similarly, ratings of greater confidence may have been in part due to social desirability effects and not fully reflective of growth in confidence. Although data collection did not occur during the heigh of COVID-19, healthcare was still significantly impacted between Fall 2022 and Spring 2024, which may have influenced participants’ clinical responsibilities, training experiences, and engagement with improvement projects. Although LHI delivery remained fully virtual and unchanged during this period, the broader healthcare context may have affected participants’ project progression (positive or negative) and confidence that cannot be determined from the current evaluation. Finally, the sample was limited to clinical and operational leaders within the VA which may limit the generalizability to other health professionals not in health system with formal opportunities for training in quality improvement and implementation science. Future work should include objective measures and the longitudinal impact of LHI on implementation success, faculty advancement, and institutional culture beyond learners within the VA. Additionally, as interest grows in embedding implementation and improvement science into medical education, LHI offers a replicable and flexible framework that can support learners across the educational continuum.

## Conclusion

LHI addresses a critical gap in healthcare improvement training by focusing on the interpersonal, strategic, and implementation skills needed to lead change effectively within complex systems. By integrating elements of leadership, team science, and implementation theory into practical, learner-centered approach, LHI is designed to build clinicians’ and operational leaders’ confidence and capacity, as change agents. As healthcare systems increasingly prioritize outcomes-driven care, educational strategies like LHI can empower frontline professionals to implement and sustain improvements will be essential. In this evaluation, participation in LHI was associated with increased self-reported confidence in healthcare improvement activities. While these findings are promising, further work is needed to assess whether gains translate into implementation project success, sustained change, and system-level impact. Broader adoption and continued evaluation of LHI may contribute meaningfully to learner engagement and the diffusion of high-quality clinical practices across diverse institutional settings.

## Supplementary Information

Below is the link to the electronic supplementary material.

Supplementary Material 1

Supplementary Material 2

## Data Availability

The data that support the findings of this study are not publicly available due to U.S. Department of Veterans Affairs (VA) policies, which prohibit the sharing of VA data outside the VA firewall. As such, these data are not available for external access.

## Abbreviations

BCM: Baylor College of Medicine
HSR&D: Health Services Research & Development
IRB: Institutional Review Board
LHI: Leading Healthcare Improvement
MEDVAMC: Michael E. DeBakey Veterans Affairs Medical Center
QI: Quality Improvement
QUERI: Veterans Affairs Quality Enhancement Research Initiative
REDCap: Research Electronic Data Capture
VA: U.S. Department of Veterans Affairs
VAQS: Veterans Affairs Quality Scholars
VHA: Veterans Health Administration
