# Supplementary material for: Leading healthcare improvement: an implementation strategy
Source: BMC Health Serv Res. 2026 Jun 30;26:1289. doi: 10.1186/s12913-026-15046-4 (PMC13599253; doi:10.1186/s12913-026-15046-4)
Supplement: Supplementary file 1 [file 12913_2026_15046_MOESM1_ESM.pdf]

LHI Pre-Evaluation

Please complete this Leading Healthcare Improvement Pre-Evaluation by Sunday, March 2nd at midnight CST. It should take you less than 5 minutes to complete the survey.

Please contact ashley.thompson14@va.gov with any questions.

What term are you registered to take this course?

- ◯ Spring
- ◯ Fall

What year are you registered to take this course?

____

Full Name

____

What is your profession? Choose all that apply

- ☐ Administration/operations
- ☐ Research (i.e., Investigator, analyst)
- ☐ Research staff (i.e., research coordinator, health science specialist)
- ☐ Project/program management
- ☐ Clinical
- ☐ Trainee
- ☐ Other

If other, please specify

____

Please select your clinical background

- ◯ Nurse
- ◯ Nurse Practitioner
- ◯ Occupational Therapist
- ◯ Pharmacist
- ◯ Physical Therapist
- ◯ Physician
- ◯ Physician Assistant
- ◯ Psychologist
- ◯ Social Worker
- ◯ Speech Language Pathologist
- ◯ Other

If other clinical background, please specify

____

What is your primary affiliation to the VA?

- ◯ VA Facility
- ◯ VISN
- ◯ Central Office
- ◯ Other

If other VA affiliation, please specify

____

Please specify the name of your facility, VISN, or Central Office affiliation.

____

"Primary Project" is defined as the project you will be working on during this course.

What is your role on the primary project?
- ◯ Team Lead
- ◯ Team Member
- ◯ Key Partner

Do you have protected time to work on the primary project?
- ◯ Yes
- ◯ No

How much protected time (per week) do you have to work on the primary project?
- ◯ Less than 1 hour
- ◯ 1-2 hours
- ◯ 2-4 hours
- ◯ 5+ hours

Approximately how many team members do you have on the primary project?
- ◯ 1-5
- ◯ 5-10
- ◯ 10-20
- ◯ 20+

What types of health professionals are on the primary project team? Choose all that apply
- ☐ Administration/Operations
- ☐ Research (i.e., Investigator, analyst)
- ☐ Research staff (i.e., research coordinator, health science specialist)
- ☐ Project/program management
- ☐ Clinical
- ☐ Trainee
- ☐ Other

If other, please specify

____

Please select your team members' clinical backgrounds. Select all that apply
- ☐ Nurse
- ☐ Nurse Practitioner
- ☐ Occupational Therapist
- ☐ Pharmacist
- ☐ Physical Therapist
- ☐ Physician
- ☐ Physician Assistant
- ☐ Psychologist
- ☐ Social Worker
- ☐ Speech Language Pathologist
- ☐ Other

If other clinical background, please specify

____

Please indicate the reach of the primary project
- ☐ Local (i.e., VA Medical Center or CBOC, clinic/unit)
- ☐ Regional (i.e., VISN)
- ☐ National

What national, regional, and/or local VA priority does your project align with?

____

What is the current status of the primary project?
[Please rate EACH STEP below, as relevant]

	Completed	In Progress	Will Complete in Future
Brainstorming Stage	○	○	○
Aim Development	○	○	○
Building Teams	○	○	○
Developing Measures	○	○	○
Pilot Testing	○	○	○
Implementation/Data Collection	○	○	○
Data Analysis	○	○	○
Evaluation/PDSA	○	○	○
Dissemination	○	○	○

Have you ever participated in a healthcare improvement or change project? (e.g. personal improvement project, workplace initiative, or other experience that used formal improvement principles and methods)?

○ Yes
○ No

Please rate your current confidence level for each of the following:

	None	Very low	Low	Moderate	High	Very high	Extremely high
Communicate the urgency of a QI project to key partners	○	○	○	○	○	○	○
Develop and lead effective, interprofessional teams	○	○	○	○	○	○	○
Create and communicate project vision	○	○	○	○	○	○	○
Develop strategies to remove barriers for project implementation and success	○	○	○	○	○	○	○
Communicate project impacts to leadership	○	○	○	○	○	○	○
