# Supplementary material for: Leading healthcare improvement: an implementation strategy
Source: BMC Health Serv Res. 2026 Jun 30;26:1289. doi: 10.1186/s12913-026-15046-4 (PMC13599253; doi:10.1186/s12913-026-15046-4)
Supplement: Supplementary file 2 [file 12913_2026_15046_MOESM2_ESM.pdf]

Entry

Please complete the survey below.

Thank you!

1) First name ______________________

2) Last name ______________________

3) Email ______________________

4) What term are you registered to take this course?
   - ◯ Spring
   - ◯ Fall

5) What year are you registered to take this course? ______________________

LHI Exit Survey

Please complete the survey below.

Thank you!

Did you attend all 6 LHI modules?	◯ Yes<br>◯ No
If no, please select the LHI modules you DID attend:	☐ Session 1 - Generate Urgency<br>☐ Session 2 - Build Teams<br>☐ Session 3 - Create Vision & Communicate<br>☐ Session 4 - Solve Problems & Celebrate Victories<br>☐ Session 5 - Build Momentum & Embed Change<br>☐ Session 6 - Learner Presentations
Please describe why you did not attend all sessions (check all that apply)	☐ Not enough protected time<br>☐ Not on my calendar/forgot<br>☐ Other professional obligation(s)<br>☐ Other personal obligation(s)<br>☐ Session topic did not seem useful/interesting<br>☐ Other
Other, please describe	__________________________________

Please rate the course on the following domains:

	Not at All	Somewhat	Mostly	Very
The learning objectives were met	◯	◯	◯	◯
The content was relevant to my practice	◯	◯	◯	◯
The workshop format was effective	◯	◯	◯	◯
The pre-work videos were effective	◯	◯	◯	◯
The tools were effective	◯	◯	◯	◯
The course coordinator and faculty were efficient and responsive	◯	◯	◯	◯
The course included sufficient interactive opportunities to answer my questions	◯	◯	◯	◯
I found it valuable to have time for faculty and learners to provide feedback during the workshop on my particular QI project	◯	◯	◯	◯

Overall Assessment

	Poor	Fair	Good	Very Good	Excellent
Overall appraisal of the course	◯	◯	◯	◯	◯

Would you recommend this course to others?

◯ Yes
◯ No

Do you have any suggested improvements to the course?

____

Do you intend to make practice change as a result of this course?

◯ Yes
◯ Possibly
◯ No

If you do not intend to make practice change(s) as a result of this course, it is because ...

◯ The course confirmed current practice
◯ I am not convinced of the need to change
◯ There are prohibitive barriers

What is the current status of your QI project? [Please rate EACH STEP below, as relevant]

	Completed	In Progress	Will Complete in Future
Brainstorming Stage	◯	◯	◯
Aim Development	◯	◯	◯
Building Team	◯	◯	◯
Developing Measures	◯	◯	◯
Pilot Testing	◯	◯	◯
Implementation/Data Collection	◯	◯	◯
Data Analysis	◯	◯	◯
Evaluation/PDSA	◯	◯	◯
Dissemination	◯	◯	◯

Please briefly describe any data or findings available for your QI project:
[Skip if not applicable]

____

Please briefly describe any presentations, accepted abstracts, submitted or accepted publications, etc., for your QI project:
[Skip if not applicable]

____

Overall, do you feel that you have the skills needed to conduct your QI project?

◯ Yes; definitely
◯ Yes; mostly
◯ Possibly; still too early to tell
◯ No
◯ Other

If No or Other, please explain:

____

What barriers did this course help you overcome in relation to your QI project, and in what way did the course facilitate this process?
[Skip if not applicable] __________________________________

Do you foresee any additional barriers to implementing your project?

	None/Minimal	Sizeable	Insurmountable
Leadership buy-in	◯	◯	◯
Culture	◯	◯	◯
Organization priority change	◯	◯	◯
Access to data	◯	◯	◯
Project scope	◯	◯	◯
Resources	◯	◯	◯
Your time availability	◯	◯	◯
Marketing of my program	◯	◯	◯

After completing the LHI training, please rate your current confidence level for each of the following:

	None	Very low	Low	Moderate	High	Very high	Extremely High
Communicate the urgency of a QI project to key partners	◯	◯	◯	◯	◯	◯	◯
Develop and lead effective, interprofessional teams	◯	◯	◯	◯	◯	◯	◯
Create and communicate project vision	◯	◯	◯	◯	◯	◯	◯
Develop strategies to remove barriers for project implementation and success	◯	◯	◯	◯	◯	◯	◯
Communicate project impacts to leadership	◯	◯	◯	◯	◯	◯	◯

Any feedback you would like to give to your coach: __________________________________

Any additional comments: __________________________________
